# Supplementary figures and images for: Enhancing portability of trans-ancestral polygenic risk scores through tissue-specific functional genomic data integration
Source: PLoS Genet. 2024 Aug 7;20(8):e1011356. doi: 10.1371/journal.pgen.1011356 (PMC11333000; doi:10.1371/journal.pgen.1011356)

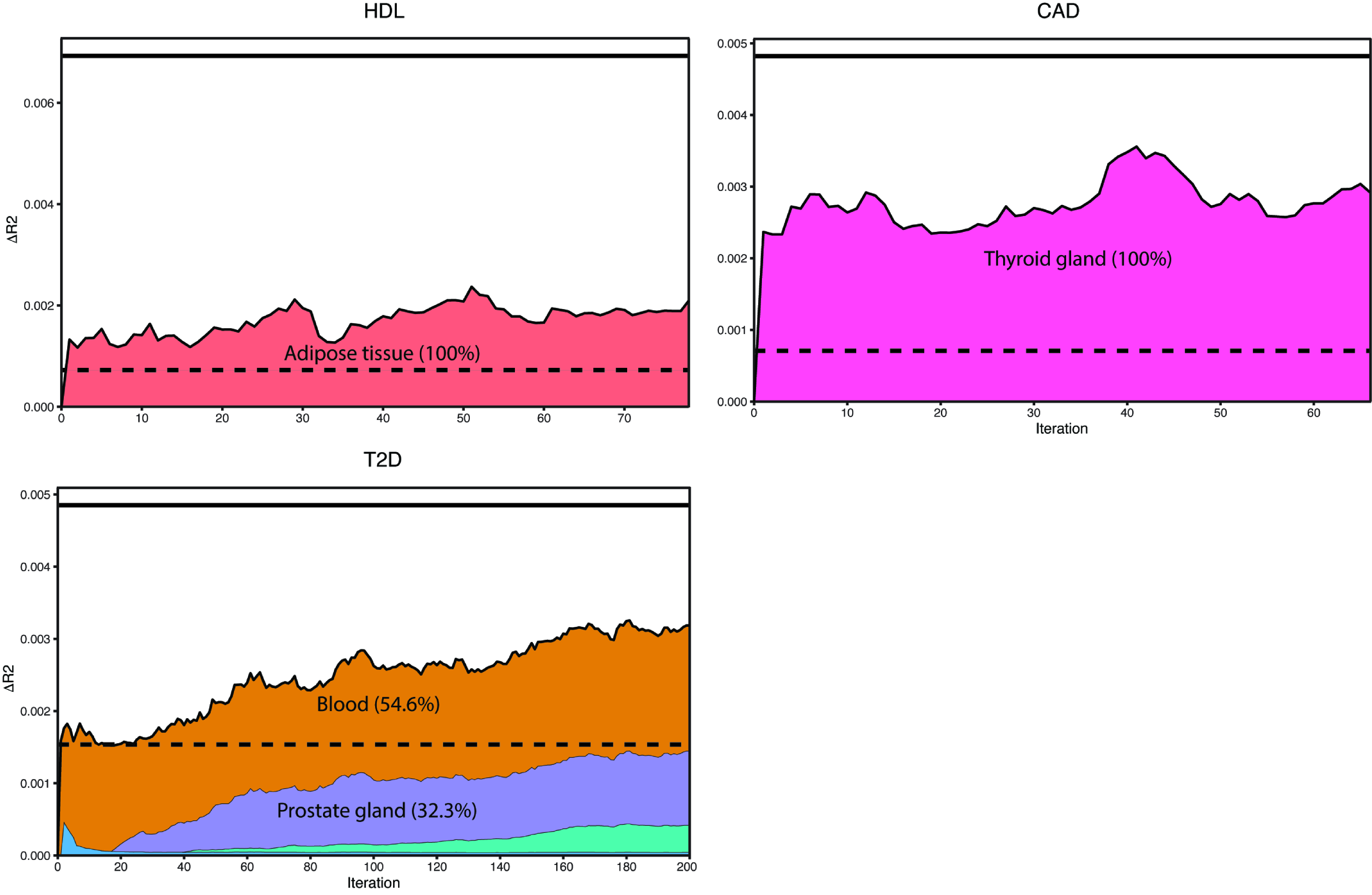

Supplement: S1 Fig — (TIF) [file pgen.1011356.s002.tif]

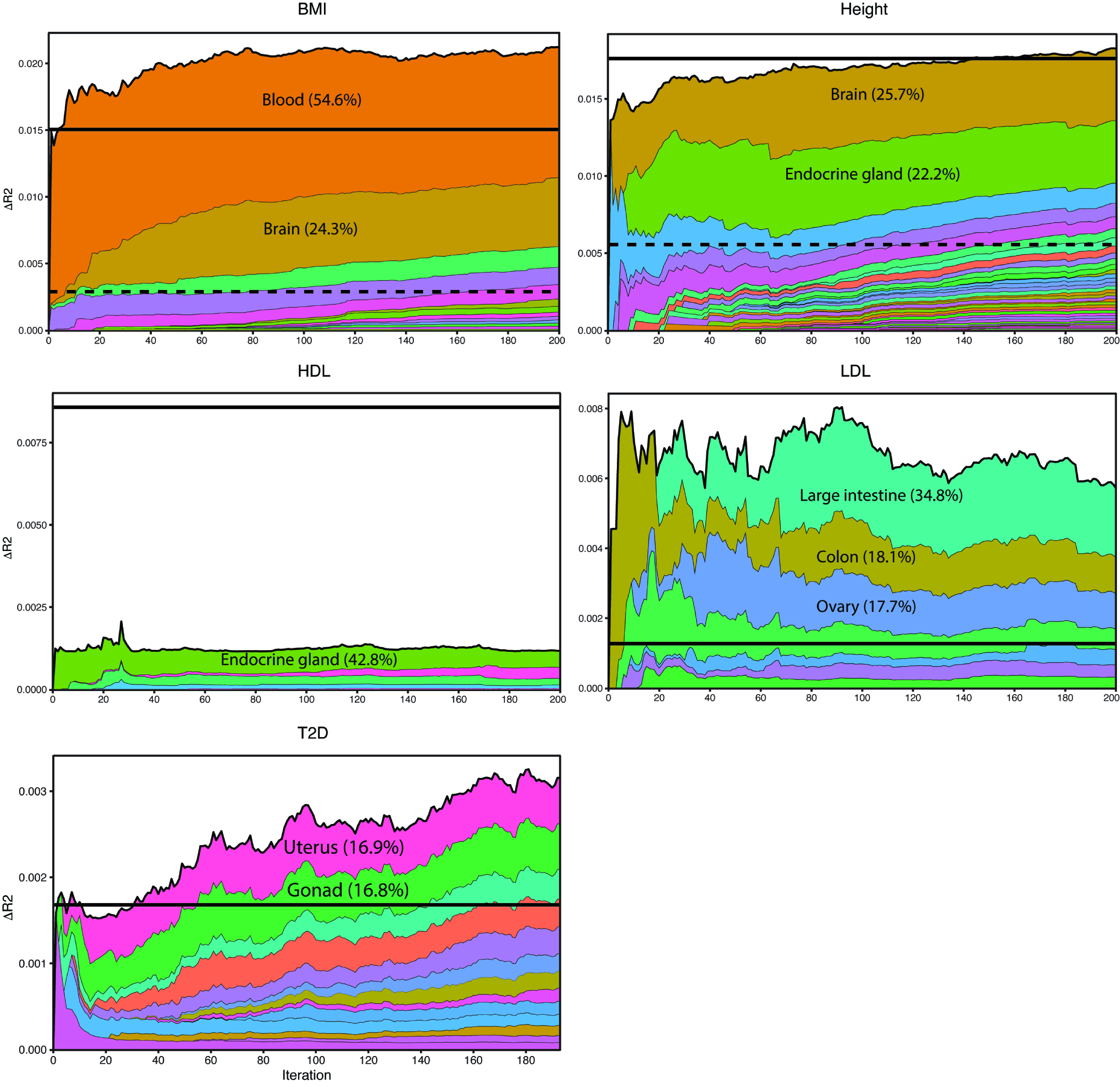

Supplement: S2 Fig — (TIF) [file pgen.1011356.s003.tif]

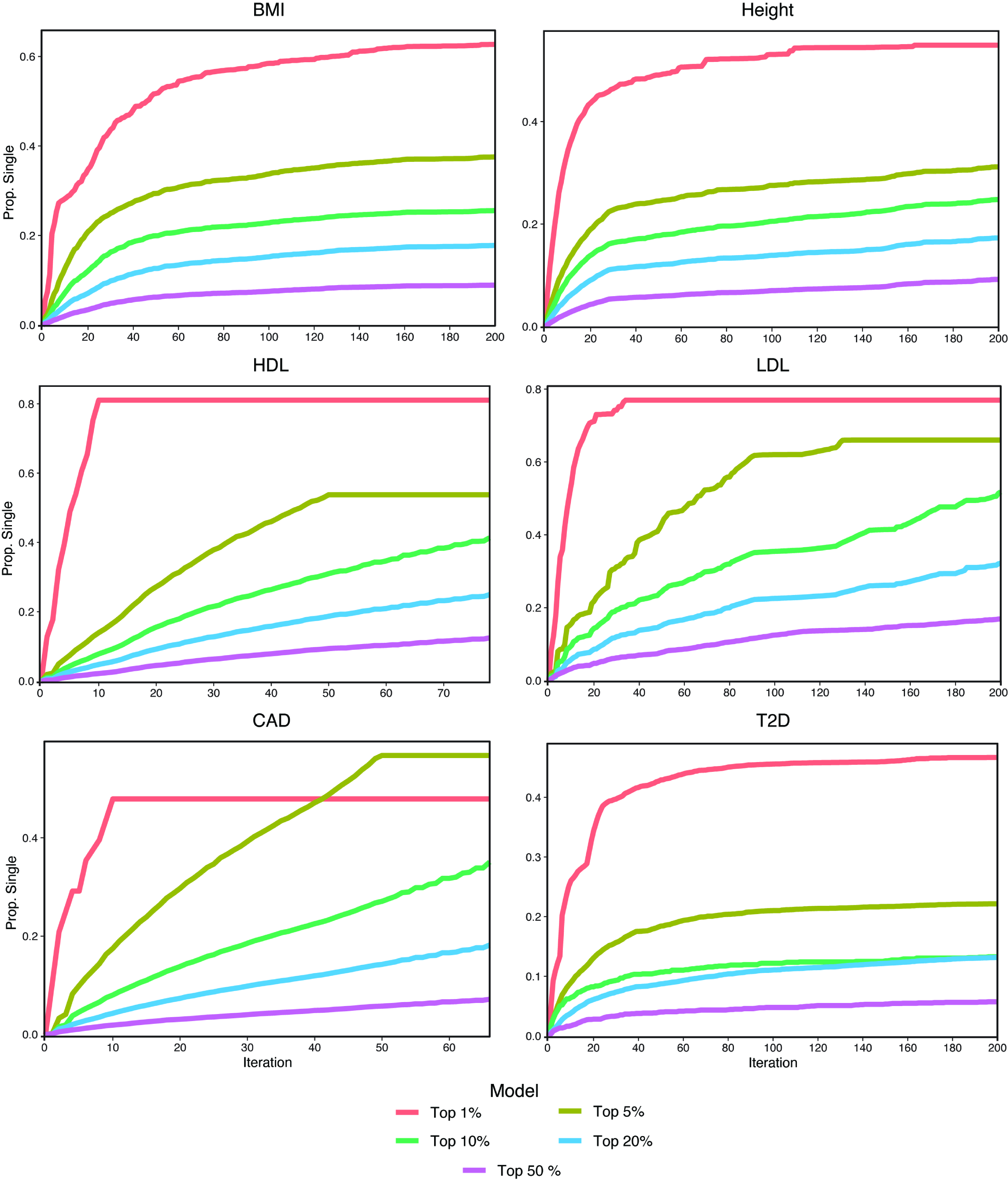

Supplement: S3 Fig — (TIF) [file pgen.1011356.s004.tif]

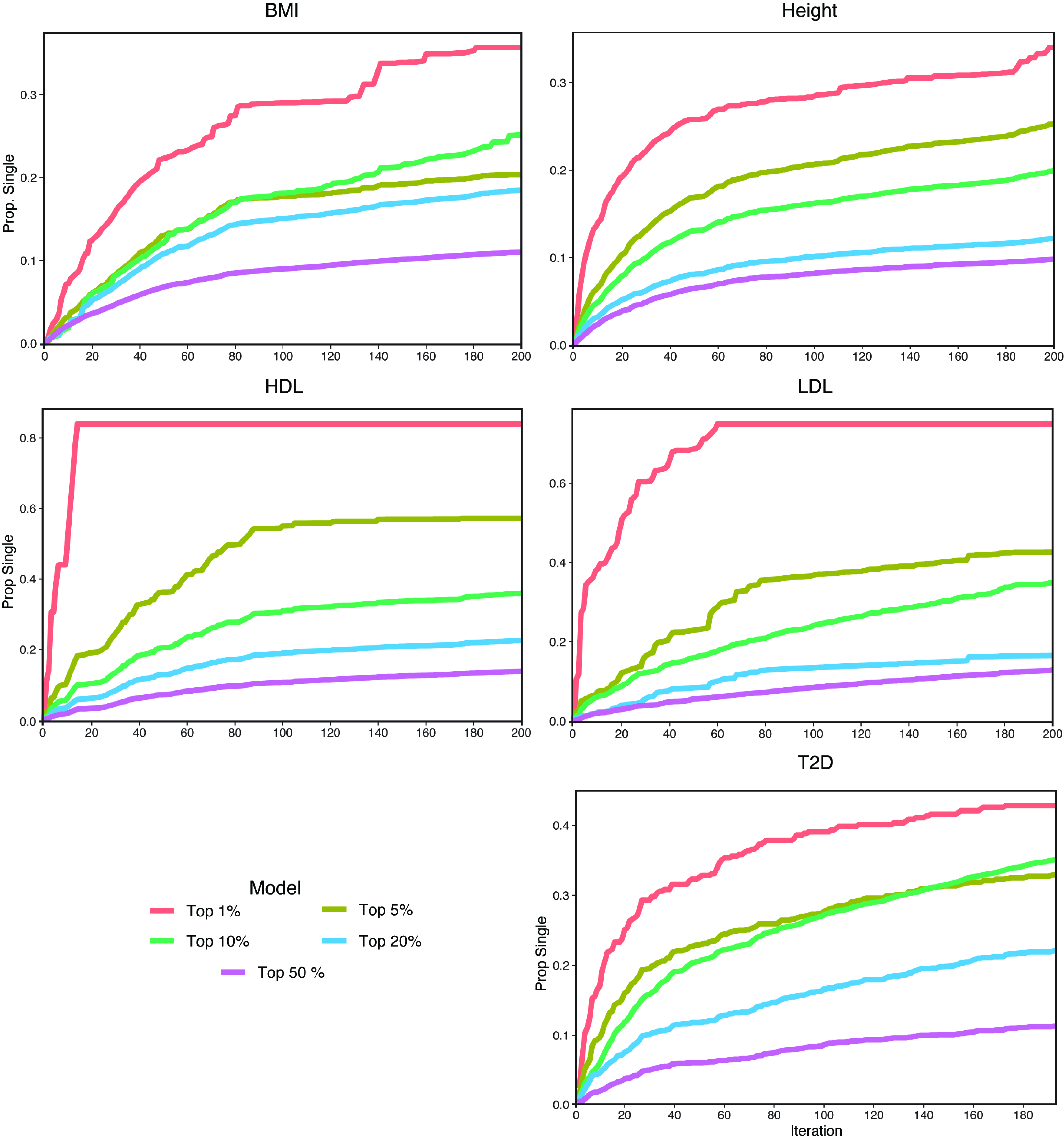

Supplement: S4 Fig — (TIF) [file pgen.1011356.s005.tif]

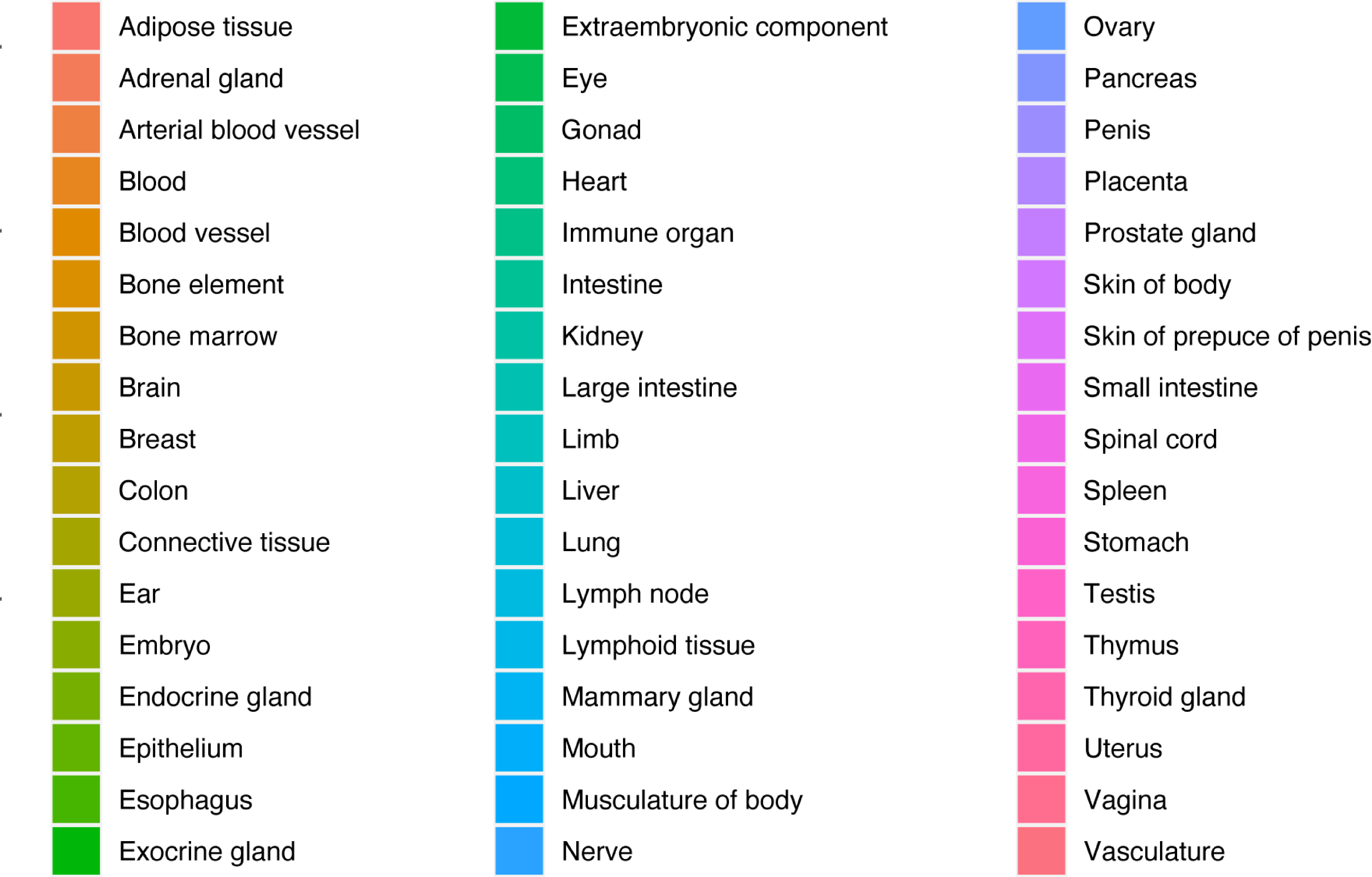

Supplement: S5 Fig — (TIF) [file pgen.1011356.s006.tif]
