# Supplementary material for: Enhancing portability of trans-ancestral polygenic risk scores through tissue-specific functional genomic data integration
Source: PLoS Genet. 2024 Aug 7;20(8):e1011356. doi: 10.1371/journal.pgen.1011356 (PMC11333000; doi:10.1371/journal.pgen.1011356)
Supplement: Attachment — Submitted filename: PRS_PGENETICS.pdf [file pgen.1011356.s013.pdf]

Review of “Enhancing Portability of Trans-Ancestral Polygenic Risk Scores through Tissue-Specific Functional Genomic Data Integration”

March, 2024

This paper aims to improve trans-ancestral portability of Polygenic Risk Scores by utilizing functional genomic data. The authors have proposed an algorithm, named as the Trans-ancestral iterative tissue refinement (TITR) and developed PRS model based on this which prioritizes functional mutations from multiple tissues. The data analysis results show an improved prediction accuracy in terms of R^2 over single tissue implementation. The paper is overall nicely written, but in general, a summary/chronological steps of the data modeling should be included for clarity. I have a few comments/questions as below:

1. Introduction section, page 2 Line 21: The authors have mentioned several competing and existing approaches. They need to better explain these approaches and drawbacks, specially, for AnnoPred.

2. GWAS summary statistics and functional genomic anotations: Can the authors provide a tabulated view of the data structure?

3. UKB genotypes section: the authors randomly selected a set of individuals for training and another set for testing. It was not clear to me, if cross validation was done? This is important to minimize selection bias.

4. For the proposed TITR algorithm, can the authors provide more specific step by step mathematical algorithm in tabulated format? The current description, while elaborate, is extremely verbose. An algorithmic structure or a table representation will be helpful for better understanding.

5. The authors should site the following two papers in context of multi-omics data integration in the introduction section:

   - "Cooperative learning for multiview analysis" - Ding et al, *PNAS*, 2022

- An integrated Bayesian framework for multi-omics prediction and classification - Mallick et al, *Statistics in Medicine*, 2023

6. Pl comment on time complexity and how the proposed method compares with the competing models.
