# Supplementary material for: Enhancing portability of trans-ancestral polygenic risk scores through tissue-specific functional genomic data integration
Source: PLoS Genet. 2024 Aug 7;20(8):e1011356. doi: 10.1371/journal.pgen.1011356 (PMC11333000; doi:10.1371/journal.pgen.1011356)
Supplement: Attachment — Submitted filename: PLOS Reviewer Responses.docx [file pgen.1011356.s014.docx]

Reviewer #1:

This paper aims to improve trans-ancestral portability of Polygenic Risk Scores by utilizing functional genomic data. The authors have proposed an algorithm, named as the Trans-ancestral iterative tissue refinement (TITR) and developed PRS model based on this which prioritizes functional mutations from multiple tissues. The data analysis results show an improved prediction accuracy in terms of R^2^ over single tissue implementation. The paper is overall nicely written, but in general, a summary/chronological steps of the data modeling should be included for clarity.

We thank the reviewer for the kind comments. To help summarize the steps of the model, we have included an additional figure (Figure 3) to the manuscript to provide a high-level overview of the major steps in TITR training and validation.

1. Introduction section, page 2 Line 21: The authors have mentioned several competing and existing approaches. They need to better explain these approaches and drawbacks, specifically, for AnnoPred.

We thank the reviewer for the suggestion and have added text to further explain approaches and drawbacks to existing models. We have edited the manuscript to include the following:

An example method is AnnoPred [(Hu et al. 2017)](https://paperpile.com/c/poUIkH/9HEr), which assesses heritability enrichment across a set of functional annotations for a given GWAS trait. These annotation-specific enrichment estimates are then implemented in a Bayesian model as a prior distribution on GWAS SNP effect sizes. AnnoPred conferred greater predictive accuracy over PRS models generated from all GWAS SNPs, genome-wide significant SNPs, SNPs prioritized by pruning and thresholding, and by LDpred [(Vilhjálmsson et al. 2015)](https://paperpile.com/c/poUIkH/kdzU), which does not prioritize functional SNPs. This shows that introducing functional evidence of regulatory activity into SNP prioritization methods results in well-powered PRS models and better isolation of causal functional SNPs at GWAS loci. However, AnnoPred relies on well-matched training and target ancestry datasets, limiting its usefulness in cross-population PRS modeling.

2. GWAS summary statistics and functional genomic annotations: Can the authors provide a tabulated view of the data structure?

In order to allow others to easily generate data in the correct formats, we have now added example data files to the git repository (<https://github.com/bcrone/TITR/>). These are in tab delimited format and contain a header line describing the structure of the files. We have also included a description of the headers and a link to the repository in the methods section of the manuscript.

3. UKB genotypes section: the authors randomly selected a set of individuals for training and another set for testing. It was not clear to me, if cross validation was done? This is important to minimize selection bias.

We appreciate the reviewer’s concern regarding selection bias in identifying training and validation samples from the UK Biobank. Training and optimizing of the TITR model targets individuals of European ancestry to isolate GWAS hits that are also functional, therefore likely commonly shared in other populations. For our purposes, we specifically identified a cohort of 40,000 unrelated white British European ancestry samples from the UK Biobank to train and optimize TITR models in Europeans. Validation of the TITR model targets individuals of non-European ancestry to evaluate how well the isolated functional GWAS hits transfer across populations. For our purposes, we specifically identified a cohort of 7,324 unrelated African ancestry samples in the UK Biobank to validate the performance of European-derived functional PRS models in non-European ancestries. We do note that a recent survey of the UK Biobank showed participation bias due to over-representation of healthy volunteers in recruited individuals (Schoeler et al. 2023), but this is certainly the case for any large cohort volunteer-based biobanks.

4. For the proposed TITR algorithm, can the authors provide more specific step by step mathematical algorithm in tabulated format? The current description, while elaborate, is extremely verbose. An algorithmic structure or a table representation will be helpful for better understanding.

We thank the reviewer’s suggestion for creating a tabular layout of the TITR algorithm, and agree that such a format would provide better understanding of the algorithm. We have added the following table to the methods section of the manuscript:

| Algorithm Step | Description | Tools/Datasets | Conditions | Output |
| --- | --- | --- | --- | --- |
| Partition *h^2^* | Estimate *h^2^* and calculate  $\tau^{*}$ for each functional annotation | Stratified LDSC  GWAS summary statistics  Functional annotation models | $\tau^{*}$ > 0  p < 0.05/51 (Bonferroni correction) | List of prioritized candidate functional annotation models |
| Calculate scores | Calculates allelic scores for lead partitions of prioritized functional annotation models in training samples | PLINK  GWAS summary statistics  UKB EUR training samples | Clump *r^2^* = 0.2  Tests 370 p-value thresholds $p\in[0.00001, 1]$ | PLINK scoring profiles for UKB EUR training samples |
| Optimize PRS | Constructs linear regression models for each candidate tissue scores, selects model with greatest $\Delta R^{2}$ over current model | Custom Python tools  PLINK scoring profiles | 1st iteration: passes ANOVA test for nested linear models  Subsequent iterations: passes Cox test for non-linear models | Master model log (tissue, partition, $\Delta R^{2}$, SNP count, etc.)  PRS model SNP list  Regression coefficients for training samples |
| Validate PRS | Generates allelic scores for AFR validation samples  Constructs linear regression models across all iterations | PLINK  Custom Python tools  GWAS summary statistics  UKB AFR validation samples | None - final model has passed all conditions in optimization | Validation model log (tissue, partition,  $\Delta R^{2}$, SNP count, etc.)  Regression coefficients for validation samples |

5. The authors should site the following two papers in context of multi-omics data integration in the introduction section:
   a. “Cooperative learning for multiview analysis” - Ding et al, *PNAS*, 2022
   b. An integrated Bayesian framework for multi-omics prediction and classification - Mallick et al, *Statistics in Medicine,* 2023

We thank the reviewer’s suggestion for additional resources in discussion of multi-omics data integration in the introduction. We have edited the manuscript to discusses these two publications:

Additional approaches in combining heterogeneous genomic information like cooperative learning models (Ding et al. 2022) and Bayesian ensemble methods (Mallick et al. 2024) can also boost signal and achieve higher predictive accuracy of functional genomic elements.

6. Pl comment on the time complexity and how the proposed method compares with the competing models.

We appreciate the reviewer’s comment regarding the time complexity of the TITR algorithm. The runtime of the algorithm is dependent on the size of the GWAS and the number of corresponding training and validation samples targeted in model development. This gives a time complexity of O(n). Wall clock runtime per iteration of TITR varied from 30 minutes to 1 hour, depending on the GWAS and training and validation sample sizes.

Reviewer #2:

I think a flowchart or visualization for the PRS construction and TITR workflow would be very helpful.

We thank the reviewer’s suggestion for a flowchart for the PRS construction and TITR workflow. We have included an additional figure (Figure 3) to the manuscript to provide a high-level overview of the major steps in TITR training and validation.

I would also like to see a comparison method that incorporates functional annotations (not just standard P+T) to assess the improvement of transferability of your method. LDPred-funct may be easy since it uses S-LDSC, which you have already calculated, or alternatively PANPRS.

We thank the reviewer’s suggestion to compare our method against alternative functional PRS models. While we do present functional prioritization results for the cell-type-specific TF IMPACT model and show significant improvement in our models, we evaluated the performance of integrating tissue specific models in LDpred-funct. We tested the LDpred-funct model leveraging the lead TURF annotation model selected by S-LDSC and constructed trans-ancestral PRS models targeting UKBB AFR ancestry samples. Two traits - BMI and HDL cholesterol - saw the greatest prediction improvements from LDpred-funct models, while the remaining traits maintained best performance by highlighting RegulomeDB-prioritized SNPs without effect size adjustment. However, the goal of our study is to specifically highlight impactful and trait-relevant sets of variants that are shared across populations rather than rescaling betas and including the full set of variants in a GWAS. A more thorough investigation is required to understand the nuances of these predictions and is outside the scope of this study.

Reviewer #3:

This study presents a new method, TITR, which leverages tissue-specific functional annotation information to increase PRS accuracy, especially to increase the accuracy of EUR GWAS based GWAS in non-EUR target data. The authors argue that TITR can improve the PRS calculated using Pruning and P-value thresholding with annotation information from existing annotations by iteratively constructing a multiple-tissue functional PRS model.

Even though this is a topic of interest in the field, I would like to suggest some comments and questions.

TITR is supposed to increase the PRS accuracy by choosing PRS more likely to be causal instead of just in LD the the causal SNPs. If this is true, TITR can also increase PRS within the EUR population. As a supplementary test, the author can also test the optimized PRS in EUR samples.

We thank the reviewer’s suggestion to assess model performance of tissue-specific functional prioritization within the same ancestry. To address this, we assessed the performance of functional PRS models in a set of UKBB samples of unrelated white British European ancestry for both single-tissue prioritization and the TITR optimized models. In single-tissue prioritization, observed that for 4 of 6 traits tested - BMI, height, HDL, and T2D - the standard P+T model significantly outperformed any of the functional prioritization models. The remaining 2 traits - LDL and CAD - confer small accuracy improvements over the baseline null model, although RegulomeDB-derived functional prioritization models still constitute a significant improvement over the standard P+T model. For TITR-optimized models, all traits exceed the performance of the top 1% of TURF and TLand models. However, no TITR-optimized model confers greater performance over the lead TURF or TLand models.

We’ve added the following note to the discussion addressing within-ancestry functional PRS performance:

This highlights a critical distinction in LD structure differences between populations. LD is greater in Europeans due to genetic bottlenecks from out-of-Africa migrations, resulting in shorter recombination time compared to African populations who have not experienced similar bottlenecks [(C. Zhang, Hansen, and Tishkoff 2022)](https://paperpile.com/c/RqWJix/3yuz). Because of stronger LD in European populations, tagging SNPs in European-derived GWAS are likely in LD with true causal SNPs, thus potentially inflating effect size estimates of tagging SNPs due to the “winner’s curse” phenomenon [(Lohmueller et al. 2003)](https://paperpile.com/c/RqWJix/0rKU). Thus, isolating causal signal with functional prioritization for within-ancestry European likely attenuates predictive accuracy as models do not incorporate inflated tagging signal. Conversely, since LD is weaker in African populations, European-derived tagging SNPs are less likely to be in LD with true causal SNPs. Therefore, functional prioritization in African-targeted PRS models could capture causal signals otherwise unaccounted for under standard P+T conditions. Overall, these results indicate standard P+T approaches select genome-wide significant variants specific to European populations and confer greater accuracy for within-population predictions, whereas functionally-informed models select variants with probable regulatory impacts shared between populations and provide stronger predictive power in trans-ancestral models.

To give a more comprehensive evaluation, the optimized PRS should also be tested in other non-EUR populations. For example, it is very convenient to test the PRS in SAS samples in UKBB.

We thank the reviewer’s suggestion to test TITR optimized PRS models in additional non-EUR populations. We evaluated the cross-population performance of TITR models in UKBB SAS samples and observed similar $\Delta R^{2}$ performance as what we reported for UKBB AFR validation. In single-tissue prioritization, RegulomeDB-derived models (SURF, TURF, TLand) significantly outperformed the $\Delta R^{2}$ performance of standard P+T models (BMI: 0.039 TURF; height: 0.0448 TURF; HDL: 0.0155 TURF; LDL: 0.00779 TLand; CAD: 0.0135 TURF; T2D: 0.0175 SURF). TITR-optimized models outperformed the top 1% TURF model for 4 of 6 traits tested (BMI, height, LDL, T2D) and outperformed the top 1% TLand models for 3 of 5 traits tested (BMI, height, T2D). Overall, tissue-specific functional prioritization in SAS validation samples conferred larger accuracy improvements over baseline null models and standard P+T as in AFR validation samples. We have added text in the results section detailing these results.

Similarly, it will be very helpful if the authors could test the method with more traits. The existing results focus on metabolic-related traits. The result of more diverse traits (e.g. cancer-related, auto-immune disease-related, neural system/ psychiatric-related) would be much appreciated.

We thank the reviewer’s suggestion to test our TITR method with more traits. We did attempt to test our model in cancer-related traits, including breast cancer and prostate cancer, and auto-immune related traits, including asthma and rheumatoid arthritis. However, when selecting samples for validation predictions, the UKBB AFR cohorts lacked sufficient case samples to produce well powered predictions.

The authors need to give some clarification/ explanation about why their null model is “a set of covariates: age, sex, the first 10 principal components, and an independently-optimized coding-region-specific PRS”, since this practice is not very common in the field at least to my best knowledge.

This is an excellent point by the reviewer. The following note has been added to the results section of the manuscript:

The motivation behind incorporating this coding score as a covariate in our baseline model is to separate out the trait heritability of regulatory regions from those of coding variants. This is required to explicitly demonstrate the added benefit of regulatory functional prioritization in the generation of our PRS models. Since our regulatory function predictions do not predict coding impact of variants and we know that coding variants drive a significant amount of heritability, failing to regress on coding signal would not allow for comparison to the standard model PRS.

Even though the R^2 increase on top of the null model R^2 shows whether the increase exists or not, it is necessary to provide the baseline value to get the idea of the relative increase, which could be of more interest when evaluating the method.

We have now included a supplementary table including these baseline estimates (Supplementary Table 6).

Besides that, I would suggest the authors also give a more commonly used set of results where the null model contains only the covariates and the PRS optimized by TITR is compared with PRS constructed using the naive P+T method. Since the main highlights of this method is the additional PRS accuracy increase gained from the iterative construction of the PRS model, I hope the author can also provide the R^2 over the iteration calculated using this more commonly used way.

As stated in the previous response to the reviewer, the motivation behind incorporating this score in the baseline model is not only to explicitly account for disease impacts by coding GWAS variants, but also to control for coding variant influences in standard P+T models to assess the contribution of non-coding regulatory variation alone. Removing this covariate from regression models would allow standard P+T model variables to account for mutations in both coding and non-coding genomic regions, resulting in greater power over the functional prioritization PRS models restricted to non-coding mutations only. This would preclude any interpretation of the improvements of our model by no longer allowing for a direct comparison of the increased power in the non-coding regions of the genome.
